# Chemogenetic Activation of ipRGCs Drives Changes in Dark-Adapted (Scotopic) Electroretinogram

**DOI:** 10.1167/iovs.16-20448

**Published:** 2016-11

**Authors:** Nina Milosavljevic, Annette E. Allen, Jasmina Cehajic-Kapetanovic, Robert J. Lucas

**Affiliations:** 1 Faculty of Biology, Medicine and Health, the University of Manchester, Manchester, United Kingdom; 2 Centre for Ophthalmology and Vision Sciences, Institute of Human Development, the University of Manchester, Manchester, United Kingdom

**Keywords:** ipRGCs, hM3Dq, photoreceptors, scotopic ERG

## Abstract

**Purpose:**

The purpose of this study was to investigate the impact of activating melanopsin-expressing intrinsically photosensitive retinal ganglion cells (ipRGCs) on dark-adapted (scotopic) electroretinograms (ERG).

**Methods:**

We used mice (*Opn4^Cre/+^*) expressing cre recombinase in melanopsin-expressing cells for a targeted gene delivery of a chemogenetic Gq-coupled receptor, hM3Dq, to ipRGCs. Intraperitoneal injection of clozapine *N*-oxide (CNO) at 5 mg/kg was used for acute activation of hM3Dq and thus excitation of ipRGCs in darkness. Dark-adapted flash ERGs were recorded across a 9-fold range of irradiances from hM3Dq *Opn4^Cre/+^* and control *Opn4^Cre/+^* mice before and after intraperitoneal injection of CNO. A- and b-wave amplitudes and implicit times and oscillatory potentials (OPs) were analyzed. Paired-flash stimuli were used to isolate cone-driven responses.

**Results:**

Clozapine *N*-oxide application suppressed a- and b-wave amplitudes of the dark-adapted ERG across the flash intensity range in hM3Dq *Opn4^Cre/+^* mice compared to control mice. Examination of the normalized irradiance-response functions revealed a shift in b-wave but not a-wave sensitivity. No changes in a- and b-wave implicit times were detected. Total OP amplitudes were also reduced in hM3Dq *Opn4^Cre/+^* mice compared to controls following CNO administration. The paired-flash method revealed reduction in both the first (rods and cones) and second (cones only) flash response.

**Conclusions:**

Acute and selective activation of ipRGCs modulates the amplitude of both a- and b-waves of the scotopic ERG, indicating that the influence of this ganglion cell class on the retinal physiology extends to the photoreceptors as well as their downstream pathways.

One of the visual system's most extraordinary feats is its ability to function across a billion-fold range of ambient illumination, from starlight to midday sun. To achieve this ability, the retina and downstream visual pathways have mechanisms in place that actively respond to the prevailing conditions. This is evident in the transition from rod- to cone-based vision, as well as sensitivity normalization of individual photoreceptors. Changes in the retinal network as a function of ambient light also optimize sensitivity or acuity according to current conditions through effects at the level of photoreceptors, inner retina, and ganglion cell layer. Such changes can be readily measured in a field potential measurement of retinal activity, the electroretinogram (ERG).^1^

An accurate independent measurement of ambient light intensity (irradiance) would be an obvious aid in defining the state of network adaptation. Intrinsically photosensitive retinal ganglion cells (ipRGCs), which express the photopigment melanopsin, encode ambient irradiance levels in their firing activity.^2–4^ Several lines of evidence link ipRGCs to regulation of the retinal network. Knocking out melanopsin in mice reduces circadian variation in cone ERG,^5^ while elimination of the M1 class of ipRGCs impairs growth of the cone ERG b-wave that is normally associated with light adaptation.^6^ Stimuli biased towards activating melanopsin reduce b-wave implicit time^7^ and b-wave amplitude^8,9^ in the light-adapted ERG in humans and mice, respectively. An open question is how far upstream in the visual pathway do these effects reach? As the ERG b-wave is a read out of bipolar cell activity, the published data indicate that ipRGCs can influence the activity of the first postreceptoral cells; however, it remains unknown whether they also target the activity of photoreceptors themselves. Light-adapted ERG do not clarify this point, as the direct read out of photoreceptor activity (the a-wave) is typically unmeasurably low amplitude. Another limitation of previous studies is that, owing to the use of bright light as a modulator of melanopsin activity, previous data have preferentially examined the influence of ipRGCs over cone pathways. ipRGCs are responsive to dim light thanks to input from rods and cones, allowing the possibility that their influence on retinal circuits extends also to rod-evoked or dark-adapted responses in the retina across lower light intensities. Therefore, two outstanding questions are: 1) Can ipRGCs modulate retinal network all the way to the photoreceptor level? 2) Can ipRGCs affect retinal response at low light levels?

Up to now, addressing these questions remained a challenge. In order to examine the ipRGCs' contribution to retinal adaptation in visually intact animals, we previously used a silent-substitution approach in which spectrally distinct stimuli were used to differentially excite melanopsin (but remain equivalent for cone photoreceptors). This experimental approach requires light-adapted conditions and so precludes its use in answering the questions posed above. To this end, we adopted a chemogenetic approach^10^ in which hM3Dq Gq-coupled receptor is exclusively expressed in ipRGCs, allowing for their selective activation upon administration of clozapine *N*-oxide (CNO). We previously used this strategy in vivo to selectively excite ipRGCs.^11^ Using that approach in this study enabled us to specifically activate ipRGCs, independently of any light stimulation, and hence examine the impact of ipRGC activity on the scotopic ERG. We found that selective activation of ipRGCs suppressed both a- and b-wave amplitude and caused a shift in b-wave sensitivity. This reveals for the first time that ipRGCs are capable of modulating retinal activity all the way to the photoreceptors, affecting both rod and cone pathways.

## Materials and Methods

### Animals

Animal care was in accordance with the UK Animals, Scientific Procedures Act of 1986,^12^ and the study was approved by the University of Manchester ethics committee. All experiments complied with the ARVO Statement for the Use of Animals in Ophthalmic and Vision Research. Animals were kept in a 12L:12D cycle at a temperature of 22°C with food and water available ad libitum. Experiments were performed in adult (3–6 months old) *Opn4^Cre/+^* mice.^13^ Unilateral intravitreal injections of AAV2-hSyn-DIO-hM3Dq-mCherry vector (2.3 × 10^13^ genomic particles/mL; the Vector Core, University of North Carolina at Chapel Hill, NC, USA) were performed as previously reported^11^ and used hyaluronan lyase and heparinase III (200 U each) to maximize retinal penetration (total volume, 2.5 μL injected over 1 minute).^14^ Control mice underwent the same procedure, with injections including glycosydic enzymes and the virus-lacking hM3Dq receptor AAV2-hSyn-DIO-mCherry vector (1.2 × 10^13^ genomic particles/mL; UNC Vector Core). Mice were allowed at least 6 weeks to recover before being used in in vivo studies. Administration of CNO (Abcam, Cambridge, UK) was performed using an intraperitoneal (ip.) route at a dose of 5 mg/kg.

### Immunohistochemistry

Immunohistochemistry was performed as previously described^11^ in retinal wholemounts in methanol-free 4% paraformaldehyde. The primary antibodies used in these studies included rabbit anti-dsRed (product 632496; 1:1000 dilution; Clontech, Saint-Germain-en-Laye, France) and chicken anti-GFP (product ab13970; 1:1000 dilution; Abcam). The secondary antibodies were Alexa 488 conjugated donkey anti-chicken (Jackson Immunoresearch, Bar Harbor, ME, USA) and Alexa 546 conjugated donkey antirabbit (Life Technologies, Corp., Carlsbad, CA, USA) at 1:200 dilution. Images were collected using a model TCS SP5 AOBS inverted confocal microscope (Leica, Wetzlar, Germany), using a 40×/0.50 plan Fluotar objective and 1.5× confocal zoom.

### Pupillometry

Pupils were filmed in the dark under infrared illumination (light-emitting diode >900 nm), using a video camera (RoleraXR; QImaging, Surrey, Canada) in unanaesthetized and gently restrained mice before and after CNO injection (5 mg/kg, ip.). Videos were analyzed using ImageJ software (http://imagej.nih.gov/ij/; provided in the public domain by the National Institutes of Health, Bethesda, MD, USA). The areas of the pupil after CNO administration were expressed relative to the area of the pupil before CNO injection. One-sample *t* test was performed to determine whether pupil constriction was significant after administration of CNO.

### Electroretinography

Electroretinograms were recorded from *Opn4^Cre/+^* mice. Anesthesia was induced with an intraperitoneal injection of ketamine (100 mg/kg; Narketan-10, 100 mg/mL; Vetoquinol, Buckingham, UK) and xylazine (10 mg/kg; Rompun; 2% w/v, Bayer, Germany). A topical mydriatic (tropicamide 1%; Chauvin Pharmaceuticals, Kingston-upon-Thames, Surrey, UK) and mineral oil (Sigma-Aldrich, Dorset, UK) were applied to the recording eye before placement of a corneal contact lens-type electrode. Mice were placed in a stereotaxic frame to keep a fixed head position; a bite bar was also used for head support and acted as a ground. A needle reference electrode (Ambu Neuroline; Ambu, Ballerup, Denmark) was inserted approximately 5 mm from the base of the contralateral eye. Electrodes were connected to a PC (Windows operating system; Microsoft, Redmond, WA, USA) through a signal conditioner (model 1902 Mark III; Cambridge Electronic Design, Cambridge, UK), which differentially amplified (×3000) and filtered (band-pass filter cutoff of 0.5–200 Hz) the signal, and a digitizer (model 1401; Cambridge Electronic Design, Cambridge, UK). Core body temperature was maintained at 37°C throughout recording with a homeothermic heat mat (Harvard Apparatus, Cambridge, UK). Heart rate was monitored during the ERG recording and found no differences between hM3Dq *Opn4^Cre/+^* and control *Opn4^Cre/+^* mice (average heart rate, 442 ± 76 and 420 ± 44 beats per second in hM3Dq and control mice, respectively; unpaired two-tailed *t* test; *P* = 0.81).

Dark-adapted ERGs were measured in response to a 15-ms flash from a warm white light-emitting diode light source (Thorlabs Inc, Newton, NJ, USA). Neutral density filters (Edmund Optics Inc., UK) placed in the light path attenuated light in 1-log-unit increments, producing corneal irradiances spanning a 9-log-unit range (6.60–14.60 log photons/cm^2^/s). Interflash intervals ranged from 1.5 seconds at the dimmest to 30 seconds at brightest intensities. The ERG a-wave amplitude and b-wave amplitudes and implicit times were measured relative to baseline values (time of flash onset). Oscillatory potentials were extracted with a band-pass filter (80–200 Hz). The peaks of the first four oscillatory potentials were measured and summed to generate the total oscillatory potential (OP) amplitude. 2-way ANOVAs were used to test for differences in a- and b-wave amplitude, latency, or total OP amplitudes, with post hoc Bonferroni corrections, to compare responses between hM3Dq and control mice, either with vehicle or CNO treatments. For a- and b-wave amplitudes and total OP amplitudes, sigmoidal dose-response curves were fitted to irradiance response relationships. An *F* test comparison was then used to determine whether functions were best fitted with the same or two separate sigmoidal curve(s).

Paired-flash ERG recording was done at the brightest light intensity (14.60 log photons/cm^2^/s), separating the two 10-ms flashes with 700-ms interstimulus interval. 2-way ANOVAs were used to test for differences in b-wave amplitudes of first and second flash, as well as in Δb-wave amplitude between first and second flash, with post hoc Bonferroni corrections, to compare responses between hM3Dq and control mice, either with vehicle or CNO treatments.

## Results

### Using Chemogenetics To Acutely and Selectively Activate ipRGCs

**Figure 1:**
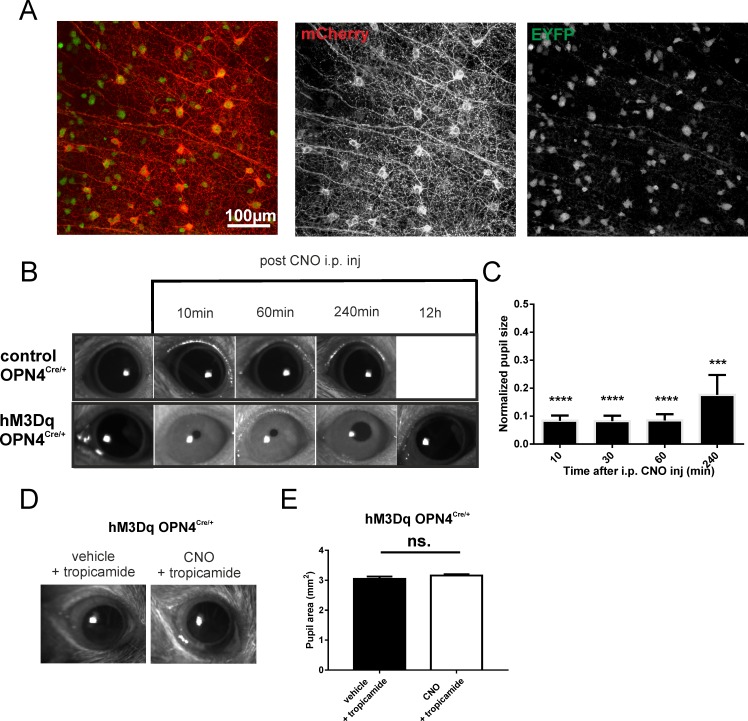
Chemogenetic activation of ipRGCs in vivo. (**A**) Exemplar of a retinal wholemount of an *Opn4Cre:Z/EYFP* mice after intravitreal injection of a viral vector (AAV2-hSyn-DIO-hM3Dq-mCherry). Immunohistochemical staining revealed hM3Dq (mCherr*y-*tag, *red channel*) expression in EYFP-positive (*green channel*) neurons, ipRGCs. (**B**) Representative images of eyes under infrared illumination from hM3Dq *Opn4^Cre/+^* and control *Opn4^Cre/+^* mice held in darkness prior to (*far left*) and at 10, 60, and 240 minutes, and 12 hours (for hM3Dq *Opn4^Cre/+^* mouse) after intraperitoneal injection of CNO (5 mg/kg). (**C**) Mean (±SEM) pupil area (normalized to pre-CNO area = 1) as a function of time after injection of CNO in hM3Dq *Opn4^Cre/+^* mice (*n* = 4, one-sample *t*-test, ****P < 0.0001, ***P < 0.001). (**D**) Representative images of an hM3Dq-injected eye treated with 1% tropicamide eye drops during ERG recordings with vehicle and CNO. (**E**) Mean (±SEM) pupil area (square millimeter) of hM3Dq-injected eyes treated with 1% tropicamide eye drops during ERG recordings with vehicle and CNO (5 mg/kg). CNO, clozapine *N*-oxide; ns, not significant.

Using viral delivery techniques, we aimed to drive targeted expression of excitatory hM3Dq in ipRGCs and thus modulate their activity in vivo (this approach is validated in reference 11). In *Opn4^Cre/+^* mice, which express cre recombinase only in melanopsin-expressing cells, we have driven cell-specific gene expression of the hM3Dq receptor via intravitreal delivery of “double-floxed” inverted open reading frame (DIO) AAV2 vector with hM3Dq-mCherry sequence. The hM3Dq receptor is a Gq-coupled receptor derived from the human muscarinic M3 acetylcholine receptor carrying two-point mutations, making it insensitive to endogenous acetylcholine but potently activated by a pharmacologically inert drug, CNO (10). Targeted expression of hM3Dq is shown in Figure 1A, where mCherry is an hM3Dq receptor's tag and GFP is a marker for ipRGCs (transduction efficiency of ∼30%).

To validate the functional consequences of hM3Dq expression in ipRGCs in vivo, we used a well-known function of ipRGC activity, the pupillary light reflex. We predicted that an intraperitoneal injection of CNO under dark-adapted conditions would result in ipRGC activation and, in turn, pupillary constriction. In mice that had received a unilateral injection of the hM3Dq-mCherry viral vector, we recorded the pupil size of injected eyes in complete darkness. We found that significant pupil constriction was detected as soon as 10 minutes after intraperitoneal injection of CNO (5 mg/kg; one-sample *t*-test; *P <* 0.0001). This effect plateaued and lasted for more than an hour (one-sample *t*-test; *P <* 0.0001), with a partial recovery ∼4 hours later (Fig. 1B, 1C). This time frame was sufficient to examine the impact of ipRGC activity on ERG recordings used in this study. No such constriction was apparent in control *Opn4^Cre/+^* (mCherry only-expressing) mice (Fig. 1B).

### Impact of Chemogenetic Activation of ipRGCs on Scotopic Flash ERG

In order to examine the effect of selective activation of ipRGCs on photoreceptors, we recorded dark-adapted flash ERGs from mice that had received unilateral intravitreal injections with virus-carrying hM3Dq-mCherry (hM3Dq *Opn4^Cre/+^* mice; *n* = 4) or mCherry sequence alone (control *Opn4^Cre/+^* animals; *n* = 5). Following 12 hours of dark adaptation, we proceeded to record dark-adapted ERGs from these mice in response to a 15-ms full-field flash across a 9-fold range of irradiances (6.60–14.60 log photons/cm^2^/s). Importantly, the pupil constriction caused by chemogenetic activation of ipRGCs was antagonized by artificially dilating the pupils with mydriatic eye drops (1% tropicamide; pupil size was measured after completion of all experiments and showed no confounding influence of pupil size on our ERG results) (Fig. 1D, 1E).

**Figure 2:**
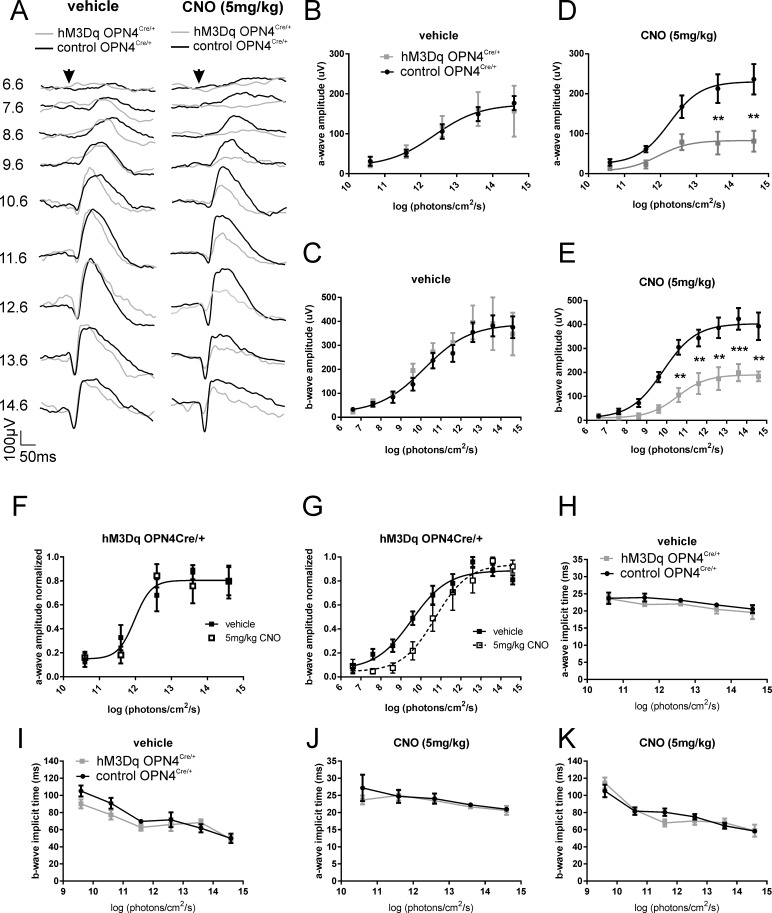
Chemogenetic activation of ipRGCs affects scotopic ERG. (**A**) Dark-adapted flash ERG recordings from representative hM3Dq *Opn4^Cre/+^* (*gray trace*) and control *Opn4^Cre/+^* (*black trace*) mice before (vehicle; *left*) and after CNO intraperitoneal injection (5 mg/kg; *right*). *Arrow* depicts flash onset. *Scale bars*: 50 ms (*x-*axis), 100 μV (*y-*axis). Numbers at *right* are total log photons/cm^2^/s. (**B**) Mean (±SEM) a-wave amplitudes from hM3Dq *Opn4^Cre/+^* (*gray squares*) and control *Opn4^Cre/+^* (*black circles*) mice before and (**D**) after CNO injection. Best fit with one curve (*F* test: *P* = 0.96) for (**B**) but not for (**D**) (*F* test: *P <* 0.0001). (**C**) Mean (±SEM) b-wave amplitudes from hM3Dq *Opn4^Cre/+^* (*gray squares*) and control *Opn4^Cre/+^* (*black circles*) mice before and (**E**) after CNO injection. Best fit with one curve (*F* test: *P* = 0.67) for (**C**) but not for (**E**) (*F* test: *P <* 0.0001). (**F**) Normalized a-wave amplitudes from hM3Dq *Opn4^Cre/+^* mice before (vehicle; *filled squares*) and after CNO injection (*open squares*) (*F* test: *P* = 0.91). (**G**) Normalized b-wave amplitudes from hM3Dq *Opn4^Cre/+^* mice before (vehicle; *filled squares*) and after CNO injection (*open squares*) (*F* test: *P* = 0.004). (**H**) Mean (±SEM) a-wave implicit time from hM3Dq *Opn4^Cre/+^* (*gray squares* and *line*) and control *Opn4^Cre/+^* (*black circles* and *line*) mice before and (**J**) after CNO injection (5 mg/kg). (**I**) Mean (±SEM) b-wave implicit time from hM3Dq *Opn4^Cre/+^* (*gray squares* and *line*) and control *Opn4^Cre/+^* (*black circles* and *line*) mice before and (**K**) after CNO injection (5 mg/kg); *n* = 4 for hM3Dq *Opn4^Cre/+^*; *n* = 5 for control *Opn4^Cre/+^* mice. 2-way ANOVA with post hoc Bonferroni corrections: *P < 0.05, **P < 0.01, ***P < 0.001. CNO, clozapine *N*-oxide.

Prior to CNO injection, both a- and b-wave amplitudes showed typical irradiance response functions (Fig. 2A–C), which were statistically similar in hM3Dq *Opn4^Cre/+^* and control *Opn4^Cre/+^* animals (a-wave: 2-way ANOVA with post hoc Bonferroni correction; *P* > 0.99; *F* test: *P* = 0.96; b-wave: 2-way ANOVA with post hoc Bonferroni correction; *P* > 0.99; *F* test: *P* = 0.67). Next, while maintaining dark-adapted conditions, we injected mice with CNO (5 mg/kg) to excite ipRGCs. Following a period of 30 minutes for stabilization/further dark adaptation, we repeated the above protocol (Fig. 2A). Following CNO injection, we found a marked difference between the ERGs recorded in hM3Dq *Opn4^Cre/+^* and control *Opn4^Cre/+^*animals. Statistical analyses revealed a significant decrease in a-wave amplitudes in hM3Dq *Opn4^Cre/+^* mice compared to that in controls injected with CNO (Fig 2D) (2-way ANOVA finds significant effect of irradiance (*P* < 0.0001), treatment (*P* < 0.0001), and interaction (*P* < 0.05); *F* test comparing sigmoidal dose response curves finds data are better fit with two separate functions; *P <* 0.0001). These effects were found to be significantly divergent at irradiances >10^13^ photons/cm^2^/s (post hoc Bonferroni correction, *P <* 0.01). Similarly, there was a significant reduction in b-wave amplitudes in hM3Dq *Opn4^Cre/+^* mice compared to controls following CNO injection (Fig. 2E) (2-way ANOVA finds significant effect of irradiance (*P* < 0.0001), treatment (*P* < 0.0001), and interaction (*P* < 0.05); *F* test, comparing sigmoidal dose response curves: *P* < 0.0001). Bonferroni post hoc analyses revealed these effects were significantly different at irradiances >10^10^ photons/cm^2^/s.

To assess whether there was also a shift in sensitivity in hM3Dq *Opn4^Cre/+^* mice upon CNO treatment, we also examined the irradiance-response curve of a- and b-wave amplitudes normalized to the maximum response (Fig. 2F, 2G). Normalized irradiance-response functions for a-wave amplitude could be fitted with the same sigmoidal curve (Fig. 2F) (*F* test, *P* > 0.05). However, those for b-wave amplitude could not (Fig. 2G) (*F* test, *P* = 0.004). Thus, although chemogenetic activation of ipRGCs had no significant impact on a-wave sensitivity, b-wave sensitivity was substantially reduced, with more than a log difference in half-maximal effective irradiance between the conditions (3.52 × 10^9^ and 5.21 × 10^10^ photons/cm^2^/s for vehicle and CNO, respectively). We also examined the effects of CNO on the timing of the ERG. Interestingly, there were no differences in a- and b-wave implicit times between conditions, either before or after CNO injection (Fig. 2G–J) (2-way ANOVA with post hoc Bonferroni correction, *P* > 0.05).

**Figure 3:**
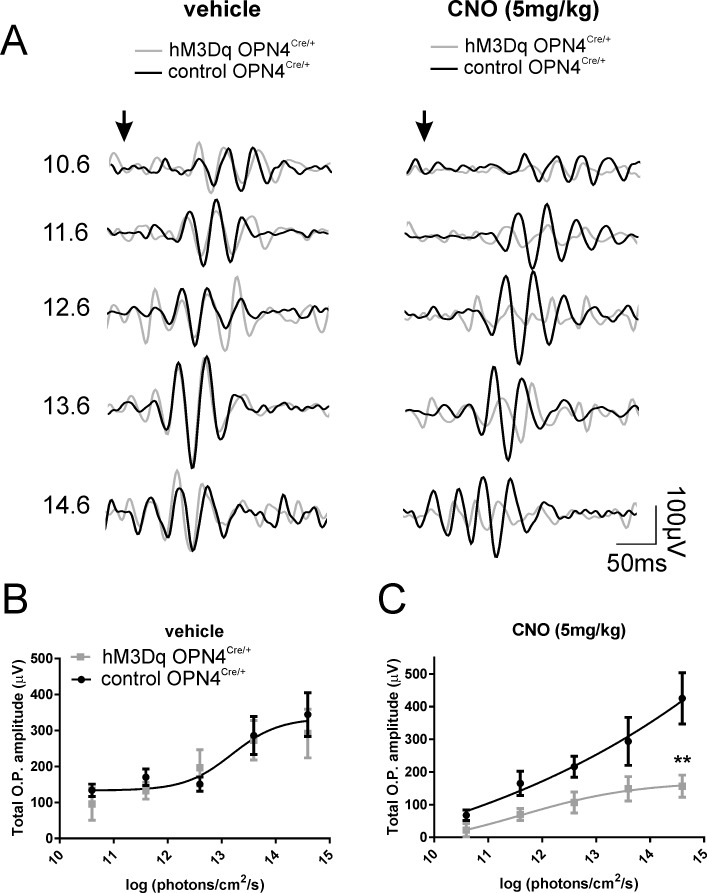
Acute activation of ipRGCs modulates OP amplitudes. (**A**) OPs extracted with a band-pass filter (80–200 Hz) from dark-adapted flash ERG recordings from representative hM3Dq *Opn4^Cre/+^* (*gray trace*) and control *Opn4^Cre/+^* (*black trace*) mice before (vehicle; *left*) and after CNO intraperitoneal injection (5 mg/kg; *right*). *Arrow* depicts flash onset. *Scale bars*: 50 ms (*x-*axis), 100 μV (*y-*axis). Numbers at *right* are total log photons/cm^2^/s. (**B**) Mean (±SEM) total OPs amplitudes from hM3Dq *Opn4^Cre/+^* (*gray squares*) and control *Opn4^Cre/+^* (*black circles*) mice before and (**C**) after CNO injection (5 mg/kg). Best fit with one curve (*F* test: *P* = 0.64) **B**, but not for **C** (*F* test: *P <* 0.005), 2-way ANOVA with post-Bonferroni corrections; *P < 0.05, **P < 0.01. CNO, clozapine *N*-oxide; OP, oscillatory potential.

Flash ERGs also drive high-frequency, low-amplitude wavelets that were superimposed on the ascending b-wave of ERG, termed OPs.^15^ Although the exact origin of OPs is still unclear, it is generally believed that they are mainly generated in the inner retina by neural interactions among bipolar, amacrine, and ganglion cells. We examined the amplitude of total OPs (sum of the first four oscillatory potentials) before (vehicle) and after CNO administration (Fig. 3A). Prior to CNO injection, total OP amplitudes were statistically similar in hM3Dq *Opn4^Cre/+^* and control *Opn4^Cre/+^* animals (2-way ANOVA with post hoc Bonferroni correction, *P* > 0.99; *F* test *P* = 0.64) (Fig. 3B). After CNO injection, we found a reduction in total OP amplitude in hM3Dq *Opn4^Cre/+^* mice compared to controls (2-way ANOVA finds significant effect of irradiance, *P <* 0.0005) and treatment (*P <* 0.0005; *F* test, *P <* 0.005) (Fig. 3C).

### Effects on Dark-Adapted Cone ERG

**Figure 4:**
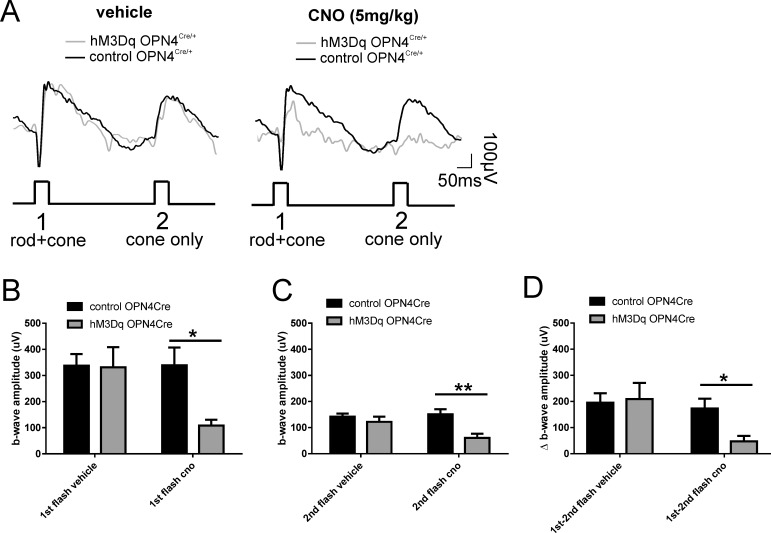
Acute activation of ipRGCs on paired flash. (**A**) Paired flash ERG recordings from representative hM3Dq *Opn4^Cre/+^* (*gray trace*) and control *Opn4^Cre/+^* (*black trace*) mice before (vehicle; *left*) and after CNO intraperitoneal injection (5 mg/kg; *right*). *Arrow* depicts flash onset. *Scale bars*: 50 ms (*x-*axis), 100 μV (*y-*axis). First flash (**1**) reflects activity of both rods and cones, and the second flash (**2**) isolates cone response. (**B**) Mean (±SEM) b-wave amplitudes from hM3Dq *Opn4^Cre/+^* (*gray bars*) and control *Opn4^Cre/+^* (*black bars*) mice before and after CNO injection (5 mg/kg) for the first (**B**) and the second (**C**) flash. (**D**) Mean (±SEM) Δb-wave amplitude between first and second flash from hM3Dq *Opn4^Cre/+^* (*gray bars*) and control *Opn4^Cre/+^* (*black bars*) mice before and after CNO injection (5 mg/kg). 2-way ANOVA with post Bonferroni corrections, *P < 0.05, **P < 0.005. CNO, clozapine *N*-oxide.

Published work provides evidence that ipRGCs regulate light-adapted cone ERG.^7,9^ To examine whether a similar influence was apparent under dark-adapted conditions, we recorded paired-flash ERGs. This approach exploits the differing recovery kinetics of rods and cones to separate their relative contribution to the ERG.^1^ Here, we presented two flashes of equal intensity (14.60 log photons/cm^2^/s) separated by 700 ms. The first flash should excite both rods and cones, but due to the poorer recovery kinetics of rods, the second flash should be predominantly cone driven, thus allowing for isolation of a cone response. Comparing the amplitude of the responses to the first and second flashes, therefore, revealed changes arising with cone or both rod and cone responses (Fig. 4A).

Prior to CNO injection, we saw a characteristic decrease in amplitude of the second flash compared to the first, consistent with the goal of isolating cone responses in the second flash (Fig. 4A). b-Wave amplitudes were statistically similar between hM3Dq *Opn4^Cre/+^* and control *Opn4^Cre/+^* animals for both first and second flashes (first flash: 2-way ANOVA with post hoc Bonferroni correction, *P* > 0.99; second flash: 2-way ANOVA with post hoc Bonferroni correction, *P* = 0.89) (Fig. 4A, 4B). Following CNO injection, for the first flash, we observed a reduction in b-wave amplitude in hM3Dq *Opn4^Cre/+^* mice compared to controls, as we observed in response to single flashes (2-way ANOVA with post hoc Bonferroni correction, *P* = 0.035) (Fig. 4A, 4B). Responses to the second flash also showed a significant difference in amplitude between hM3Dq *Opn4^Cre/+^* and control *Opn4^Cre/+^* mice (2-way ANOVA with post hoc Bonferroni correction, *P* = 0.004), indicating an influence of CNO on cone pathways (Fig. 4A, 4C). In addition, we analyzed the rod response by assessing the differences in b-wave amplitude between first and second flash. Unsurprisingly, following CNO injection, we observed a significant reduction of Δb-wave amplitude in hM3Dq *Opn4^Cre/+^* mice compared to control animals (2-way ANOVA with post hoc Bonferroni correction, *P* = 0.025). Excitation of ipRGCs thus has substantial effects on the activity of both rod and cone pathways.

## Discussion

By combining a targeted chemogenetic approach with electroretinography, we were able to examine the impact of selective activation of ipRGC activity on upstream retinal neurons under dark-adapted conditions. We found that the amplitude, but not timing, of photoreceptor (a-wave), bipolar cell (b-wave), and inner-retinal responses (OPs) were all reduced by acutely and selectively exciting ipRGCs. We further showed that selective activation of ipRGCs adjusted the amplitude of both rod- and cone-derived ERGs.

The reduction in amplitude of the scotopic a-wave raises the exciting possibility that the influence of ipRGCs extends as far as rod (and maybe cone) photoreceptors. This is the first indication that ipRGCs could impact activity of the outer retina. The a-wave is typically regarded as reflecting the activity of the photoreceptors and, although a change in photoreceptor activity it is not the only plausible event that could reduce a-wave amplitude, is the most straightforward. If we accept that the a-wave reflects photoreceptor hyperpolarization and that the efficiency of photon capture is constant, then activation of ipRGCs could produce a smaller a-wave by either raising the floor or lowering the ceiling of the photoreceptor response. In other words, the influence of ipRGCs could be reducing the dark current (e.g., lowering resting membrane potentially by closure of cGMP-gated cation channels in the outer segment and/or the activity of a Na^+^/Ca^2+^,K^+^ (NCKX) exchange in the outer-segment or the activity of Na^+^/K^+^ pumps in the inner segment), or alternatively, by decreasing the magnitude of light response (by modulating the activity of some aspect of the phototransduction cascade).^16–18^ Interestingly, a very recent study showed that rod ion conductance is modulated by melanopsin phototransduction.^19^ In the mouse, under dark-adapted conditions and across the sensitivity range we used, the flash ERG is dominated by rods. Our data therefore imply an effect on rod photoreceptors, although a similar impact on cones is not excluded.

Given that chemogenetic activation of ipRGCs modulates the activity of photoreceptors, it is not surprising that we also saw significant reductions in the amplitude of the b-wave and OPs. Thus, the most parsimonious explanation for these latter effects is that they are inherited from the reduced activity of the photoreceptors themselves. This could explain the reduced amplitude of the single-flash b-wave and OPs, although we noted that there was no change in a-wave sensitivity. Similarly, as we do not know whether the cone flash response is impacted in the same way by ipRGCs, it is not clear whether changes in the outer retina could also explain the reduced cone b-wave in our paired flash protocol. It thus remains entirely possible that ipRGCs impact the retinal network at multiple locations. ipRGC activation has been shown previously to modulate the light-adapted cone b-wave amplitude in mice^8,9^ and implicit time in humans.^7^ Allen et al.^9^ found that the reduction in b-wave amplitude was correlated with a reduction in response amplitude in the mouse dorsal lateral geniculate nucleus (dLGN) in response to a full field flash. That result was explained by a change in spatial frequency tuning of a subset of dLGN neurons, which shifted to prefer higher spatial frequencies. That study proposed that the change in b-wave amplitude may therefore reflect the same event and be a consequence of more complex changes in the retinal network. In a similar way, the origins of the changes in these downstream responses we observe here could also have more complex origins that relate to higher-level retinal processes.

Identifying the route(s) by which ipRGCs influence retinal physiology to produce these changes in the ERG is a fruitful area of future research. There are a number of examples of ipRGCs contacting other retinal neurons, both chemically and electrically.^6,20–24^ However, to date this has been restricted to contacts with inner retinal neurons suggesting that such direct contacts are not sufficient to produce the range of effects we observe. An alternative explanation for our observed effects is a more global impact on the neuromodulatory environment of the retina, such as changes in inhibitory neuromodulators, signals of light adaptation, or even changes in the retinal pH, that could extend up to the photoreceptor level.^25,26^

Although the intrinsic light response of ipRGCs (driven by melanopsin) has a low sensitivity, ipRGCs also receive signals from rods and cones.^2^ In this regard, it is perfectly feasible for ipRGCs to provide dynamic modulation of rod function even though this should occur over irradiances below the sensitivity threshold of melanopsin. The scotopic ERG is dominated by rods, especially at low flash intensities. The differences in ERG amplitude we saw here across the intensity range thus represent strong evidence that rod pathways are indeed targets of ipRGC signaling. Our paired flash protocol confirms that cone pathways are also affected (although in the absence of a measurable a-wave we cannot be sure that the effects extend to cone photoreceptors themselves). One is tempted to conclude that ipRGCs drive progressive changes in retinal circuits across a wide range of background light intensities. However, the chemogenetic manipulation, while allowing analytical examination of ipRGCs' influence on the retinal network, also leaves the retina in an unnatural state in which ipRGCs are highly excited but the rest of the retina is kept under dark-adapted conditions. It may thus be that the changes we observe are reflections of the similar shift in the retinal physiology occurring in the natural conditions over a more limited range of irradiances.

To summarize, our data add to the growing body of evidence that ipRGCs and melanopsin drive modulatory effects within the retina according to their independent measure of brightness. Using a chemogenetic approach, we have for the first time been able to reveal the impact of those effects in the scotopic ERG, demonstrating that the changes in retinal activity extend up to the photoreceptor level.

## References

[1] Weymouth AE, Vingrys AJ. Rodent electroretinography: methods for extraction and interpretation of rod and cone responses. *Prog Retin Eye Res*. 2008; 27: 1– 44. 18042420 10.1016/j.preteyeres.2007.09.003

[2] Dacey DM, Liao HW, Peterson BB, Melanopsin-expressing ganglion cells in primate retina signal colour and irradiance and project to the LGN. *Nature*. 2005; 433: 749– 754. 15716953 10.1038/nature03387

[3] Tu DC, Zhang D, Demas J, Physiologic diversity and development of intrinsically photosensitive retinal ganglion cells. *Neuron*. 2005; 48: 987– 999. 16364902 10.1016/j.neuron.2005.09.031

[4] Berson DM. Strange vision: ganglion cells as circadian photoreceptors. *Trends Neurosci*. 2003; 26: 314– 320. 12798601 10.1016/S0166-2236(03)00130-9

[5] Barnard AR, Hattar S, Hankins MW, Lucas RJ. Melanopsin regulates visual processing in the mouse retina. *Curr Biol*. 2006; 16: 389– 395. 16488873 10.1016/j.cub.2005.12.045

[6] Prigge CL, Yeh PT, Liou NF, M1 ipRGCs influence visual function through retrograde signaling in the retina. *J Neurosci*. 2016; 36: 7184– 7197. 27383593 10.1523/JNEUROSCI.3500-15.2016 PMC4938862

[7] Hankins MW, Lucas RJ. The primary visual pathway in humans is regulated according to long-term light exposure through the action of a nonclassical photopigment. *Curr Biol*. 2002; 12: 191– 198. 11839270 10.1016/s0960-9822(02)00659-0

[8] Allen AE, Lucas RJ. Using silent substitution to track the mesopic transition from rod- to cone-based vision in mice. *Invest Ophthalmol Vis Sci*. 2016; 57: 276– 287. 26818794 10.1167/iovs.15-18197

[9] Allen AE, Storchi R, Martial FP, Melanopsin-driven light adaptation in mouse vision. *Curr Biol*. 2014; 24: 2481– 2490. 25308073 10.1016/j.cub.2014.09.015 PMC4228053

[10] Armbruster BN, Li X, Pausch MH, Herlitze S, Roth BL. Evolving the lock to fit the key to create a family of G protein-coupled receptors potently activated by an inert ligand. *Proc Natl Acad Sci U S A*. 2017; 104: 5163– 5168. 10.1073/pnas.0700293104 PMC1829280 17360345

[11] Milosavljevic N, Cehajic-Kapetanovic J, Procyk CA, Lucas RJ. Chemogenetic activation of melanopsin retinal ganglion cells induces signatures of arousal and/or anxiety in mice. *Curr Biol*. 2016; 26: 2358– 2363. 27426512 10.1016/j.cub.2016.06.057 PMC5026697

[12] Inutsuka A, Yamanaka A. The physiological role of orexin/hypocretin neurons in the regulation of sleep/wakefulness and neuroendocrine functions. *Front Endocrinol (Lausanne)*. 2013; 4: 18. 23508038 10.3389/fendo.2013.00018 PMC3589707

[13] Ecker JL, Dumitrescu ON, Wong KY, Melanopsin-expressing retinal ganglion-cell photoreceptors: cellular diversity and role in pattern vision. *Neuron*. 2010; 67: 49– 60. 20624591 10.1016/j.neuron.2010.05.023 PMC2904318

[14] Cehajic-Kapetanovic J, Le Goff MM, Allen A, Lucas RJ, Bishop PN. Glycosidic enzymes enhance retinal transduction following intravitreal delivery of AAV2. *Mol Vis*. 2011; 17: 1771– 1783. 21750604 PMC3133842

[15] Wachtmeister L. Oscillatory potentials in the retina: what do they reveal. *Prog Retin Eye Res*. 1998; 17: 485– 521. 9777648 10.1016/s1350-9462(98)00006-8

[16] Bauer PJ. The complex of cGMP-gated channel and Na+/Ca2+, K+ exchanger in rod photoreceptors. *Adv Exp Med Biol*. 2002; 514: 253– 274. 12596926

[17] Schnetkamp PP. The SLC24 Na+/Ca2+-K+ exchanger family: vision and beyond. *Pflugers Arch*. 2004; 447: 683– 688. 14770312 10.1007/s00424-003-1069-0

[18] Fu Y, Yau KW. Phototransduction in mouse rods and cones. *Pflugers Arch* 2007; 454: 805– 819. 17226052 10.1007/s00424-006-0194-y PMC2877390

[19] Berkowitz BA, Schmidt T, Podolsky RH, Roberts R. Melanopsin phototransduction contributes to light-evoked choroidal expansion and rod L-type calcium channel function in vivo. *Invest Ophthalmol Vis Sci*. 2016l; 57: 5314– 5319. 27727394 10.1167/iovs.16-20186 PMC5063053

[20] Muller LP, Do MT, Yau KW, He S, Baldridge WH. Tracer coupling of intrinsically photosensitive retinal ganglion cells to amacrine cells in the mouse retina. *J Comp Neurol*. 2010; 518: 4813– 4824. 20963830 10.1002/cne.22490 PMC2967574

[21] Reifler AN, Chervenak AP, Dolikian ME, All spiking sustained ON displaced amacrine cells receive gap-junction input from melanopsin ganglion cells. *Curr Biol*. 2015; 25: 2763– 2773. 26441349 10.1016/j.cub.2015.09.018 PMC4631663

[22] Zhang DQ, Wong KY, Sollars PJ, Berson DM, Pickard GE, McMahon DG. Intraretinal signaling by ganglion cell photoreceptors to dopaminergic amacrine neurons. *Proc Natl Acad Sci U S A*. 2008; 105: 14181– 14186. 18779590 10.1073/pnas.0803893105 PMC2544598

[23] Zhang DQ, Belenky MA, Sollars PJ, Pickard GE, McMahon DG. Melanopsin mediates retrograde visual signaling in the retina. *PLoS One*. 2012; 7: e42647. 22880066 10.1371/journal.pone.0042647 PMC3411794

[24] Joo HR, Peterson BB, Dacey DM, Hattar S, Chen SK. Recurrent axon collaterals of intrinsically photosensitive retinal ganglion cells. *Vis Neurosci*. 2013; 30: 175– 182. 23834959 10.1017/S0952523813000199 PMC4316817

[25] Hirasawa H, Yamada M, Kaneko A. Acidification of the synaptic cleft of cone photoreceptor terminal controls the amount of transmitter release thereby forming the receptive field surround in the vertebrate retina. *J Physiol Sci*. 2012; 62: 359– 375. 22773408 10.1007/s12576-012-0220-0 PMC10717482

[26] Wang TM, Holzhausen LC, Kramer RH. Imaging an optogenetic pH sensor reveals that protons mediate lateral inhibition in the retina. *Nat Neurosci*. 2014; 17: 262– 268. 24441679 10.1038/nn.3627 PMC3985427

